# Supplementary material for: Differential Impact of IL-32 Isoforms on the Functions of Coronary Artery Endothelial Cells: A Potential Link with Arterial Stiffness and Atherosclerosis
Source: Viruses. 2023 Mar 8;15(3):700. doi: 10.3390/v15030700 (PMC10052544; doi:10.3390/v15030700)
Supplement: Supplementary file 1 [file viruses-15-00700-s001.zip › viruses-2219572-supplementary.pdf]

Differential impact of IL-32 isoforms on the functions of coronary artery endothelial cells: A potential link with arterial stiffness and atherosclerosis

Rémi Bunet [1,2], Marie-Hélène Roy-Cardinal [3], Hardik Ramani [1,2], Aurélie Cleret-Buhot [2], Madeleine Durand [2,4], Carl Chartrand-Lefebvre [2,5], Jean-Pierre Routy [6], Réjean Thomas [7], Benoît Trottier [8], Petronela Ancuta [1,2], David B. Hanna[9], Alan L. Landay [10], Guy Cloutier [3,5,11], Cécile L. Tremblay [1,2,†,*] and Mohamed El-Far [2,†,*]

[1] Département de Microbiologie, Infectiologie et Immunologie, Faculté de Médecine, Université de Montréal, Montréal, H3C 3J7, QC, Canada,
[2] Centre de Recherche du Centre Hospitalier de l'Université de Montréal (CRCHUM), Montréal, H2X 0A9, QC, Canada,
[3] Laboratory of Biorheology and Medical Ultrasonics, Centre de Recherche du Centre Hospitalier de l'Université de Montréal (CRCHUM), Montréal, H2X 0A9, QC, Canada,
[4] Département de Médecine, Faculté de Médecine, Université de Montréal, H3T 1J4, QC, Canada
[5] Département de Radiologie, Radio-oncologie et Médecine Nucléaire, Faculté de Médecine, Université de Montréal, Montréal, H3C 3J7, QC, Canada,
[6] Chronic Viral Illness Service, division of hematology, McGill University Health Centre, Montréal and Research Institute of McGill University Health Centre, Montréal, H4A 3J1, QC, Canada
[7] Clinique médicale l'Actuel, Montréal, H2L 4P9, QC, Canada,
[8] Centre de médecine urbaine du Quartier latin, Montréal, H2L 4E9, QC, Canada,
[9] Department of Epidemiology and Population Health, Albert Einstein College of Medicine, Bronx, NY 10461, USA,
[10] Department of Internal Medicine, Rush University Medical Center, Chicago, 60612, IL, United States
[11] Institut de génie biomédical, Université de Montréal, Montréal, H3T 1J4, QC, Canada,
† These authors contributed equally to this work.
* Correspondence: c.tremblay@umontreal.ca (C.L.T.); mohamed.el.far.chum@ssss.gouv.qc.ca (M.E.-F.)

Supplemental materials

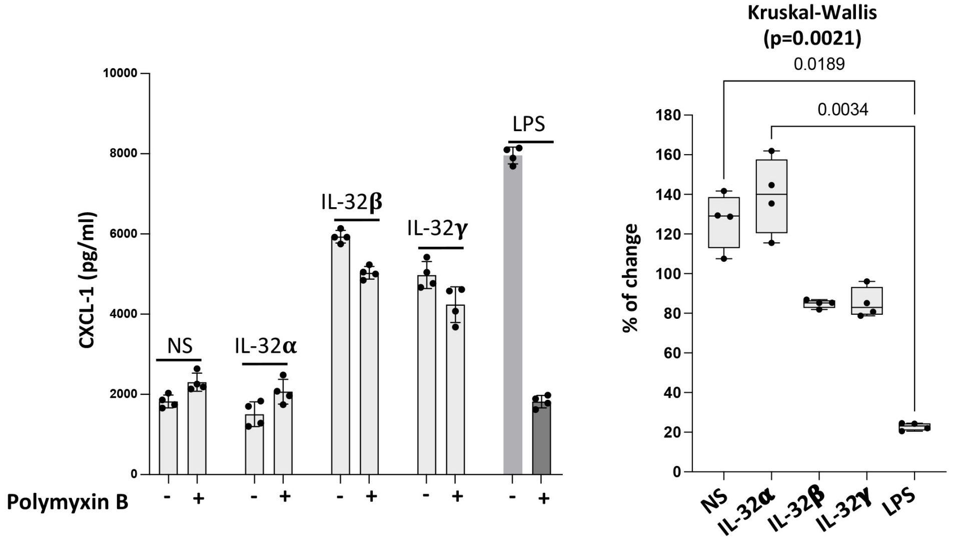

Supplemental Figure S1: Impact of Polymyxin B on CAEC. CXCL-1 expression by CAEC following stimulation with IL-32 isoforms (500ng/ml) or LPS (500ng/ml) in the presence or absence of Polymyxin B. Left panel: CXCL-1 levels in pg/ml. Right panel: Percentage of change (increase or decrease) in CXCL-1 expression for each condition (Non-stimulated NS, IL-32α-, IL-32β-, IL-32γ- and LPS-stimulated cells) from the left panel generated by the ratio between Polymyxin B treated to untreated condition. NS: non-stimulated.

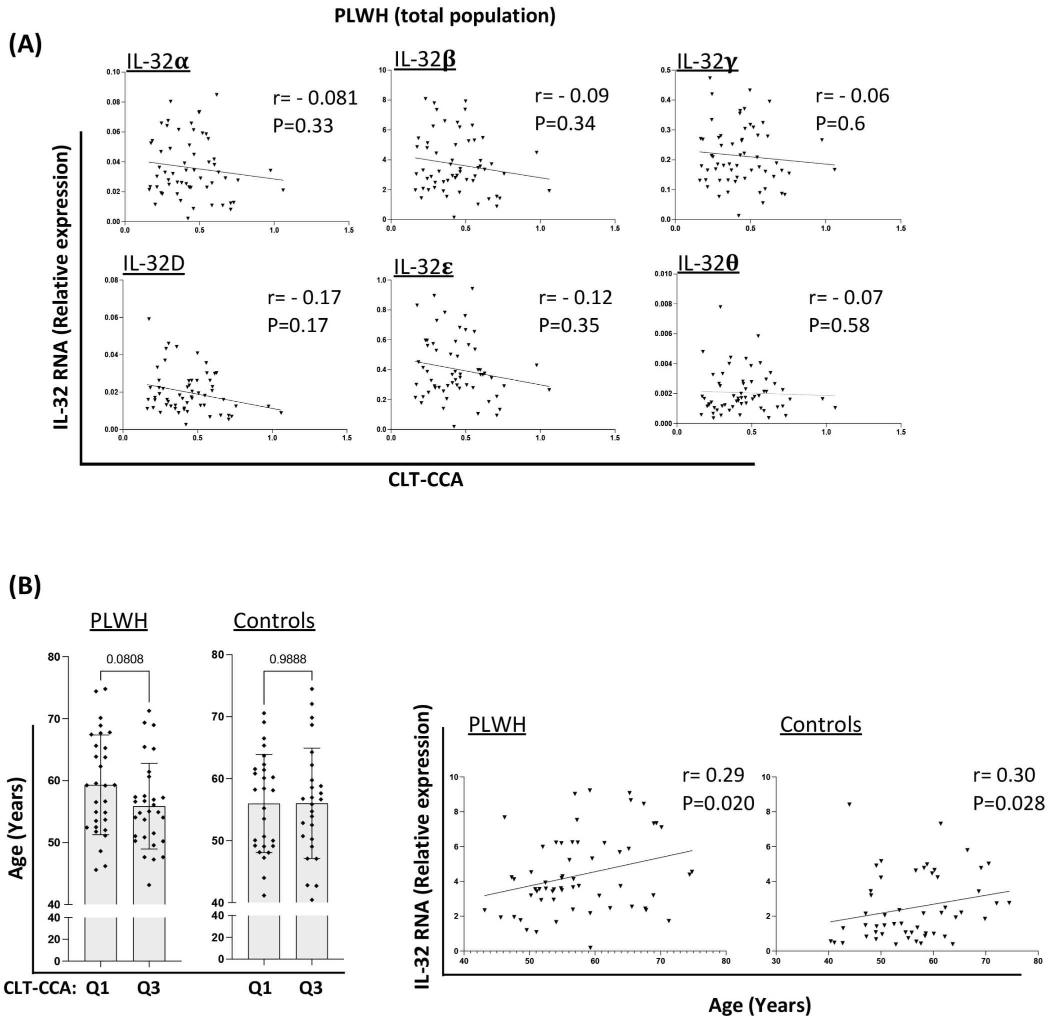

Supplemental Figure S2: A) Association between IL-32 expression and carotid artery wall stiffness in the total PLWH population**. B)** Age comparison within the lower (Q1) and upper (Q3) quartiles of CLT-CCA of PLWH (n=30 *per* quartile) and controls (n=27 for Q1 and n=26 for Q3), Left panels. Right panel: correlations between total IL-32RNA from PLWH (n=60, left panel) and controls (n=53, right panel) and age. Q1: First quartile, Q3: Third quartile. CLT: Cumulated lateral translation in common carotid artery.
